# Structural Damage Identification Based on AR Model with Additive Noises Using an Improved TLS Solution

**DOI:** 10.3390/s19194341

**Published:** 2019-10-08

**Authors:** Cai Wu, Shujin Li, Yuanjin Zhang

**Affiliations:** 1 School of Civil Engineering and Architecture, Wuhan University of Technology, Luoshi Road No.122, Wuhan 430070, China; wucai@whut.edu.cn (C.W.); sjli@whut.edu.cn (S.L.); 2 School of Safety Science and Emergency Management, Wuhan University of Technology, Luoshi Road No.122, Wuhan 430070, China

**Keywords:** damage identification, auto-regressive model, total least-squares method

## Abstract

Structural damage is inevitable due to the structural aging and disastrous external excitation. The auto-regressive (AR) based method is one of the most widely used methods for structural damage identification. In this regard, the classical least-squares algorithm is often utilized to solve the AR model. However, this algorithm generally could not take all the observed noises into account. In this study, a partial errors-in-variables (EIV) model is used so that both the current and prior observation errors are considered. Accordingly, a total least-squares (TLS_E_) solution is introduced to solve the partial EIV model. The solution estimates and accounts for the correlations between the current observed data and the design matrix. An effective damage indicator is chosen to count for damage levels of the structures. Both mathematical and finite element simulation results show that the proposed TLS_E_ method yields better accuracy than the classical LS method and the AR model. Finally, the response data of a high-rise building shaking table test is used for demonstrating the effectiveness of the proposed method in identifying the location and damage degree of a model structure.

## 1. Introduction

Civil structures are subject to many adverse factors [1,2,3,4] such as corrosion, aging, over-capacity loads, and may occasionally experience natural disasters such as earthquakes and hurricanes, and even extreme manmade events including vehicle-structure collision and explosion, causing structural damage to different degrees. In recent years, structural damage detection and identification have received much attention and different approaches have been developed [5,6]. For example, the sensor-based approaches can offer detailed information about local damage [7]. Vision and thermal-image based methods can present structural surface damage [8]. Vibration-based approaches are effective in detecting the damage in the entire structures [9]. 

Vibration-based approaches have been widely applied to identify various types of damage in real and laboratory structures owing to their noninvasive characteristics [10]. For example, Gul [11] investigated the different damage detection methods for global condition assessment of structures based on vibration data. Morita et al. Datteo et al. [12] identified the vibration responses of a real structure in Italy under operational conditions. Though various vibration-based methods, such as time-series analysis, wavelet analysis, and neural network, [10], the statistical time-series methods form an important, rapidly evolving category. Fassois and Sakellariou [13] summarized the principles and techniques of time-series methods for fault detection, identification, and estimation in vibrating structures. The time-series methods are originated in statistics and refer to a time-ordered sequence of random (stochastic) scalar or vector observations. These methods appear promising as they tend to be more accessible and less expensive than many other alternatives. The process of time-series methods involves the observation of a structure over time using periodical measurements, the extraction of damage sensitive quantities (features) from these measurements, and the statistical analysis of these quantities in order to determine the current structural state [14].

A widely used time series method is the Auto-Regressive (AR) model [15]. The AR model tries to account for the correlations between the current time parameter with the predecessors in time series, where the output variable depends linearly on its previous values and a stochastic term [16]. From the perspective of a structural engineer, a structure can be assumed as a system that contains segments such as mass, damping, and stiffness. Therefore, the AR model parameters of a structure always stay the same. When the structure is damaged, the AR parameters of these models change accordingly and become different from those in the undamaged stage. Hence the changes of AR parameters can reflect the structural inner damage. Among all the possible strategies for AR modeling, the use of classical AR models is widespread. Basically, this model assumes that there are only errors in the current observations, and the noise variance is assumed as known, for example, the Gaussian white noise. The AR parameters are most commonly estimated by the least-squares (LS) method, and have been investigated in a number of studies [15]. Methods such as Mahalanobis Squared Distance [17], Yule-Walker equations [18] and the maximum-likelihood method [19] have also been adopted to solve the AR model. 

Since the AR data are determined from measurements, there may be noises not only in the current observations but also in the predecessors. Hence, the classical AR model would be, inaccurately [20]. The biases of the classical AR model are proved in some researches [21,22]. For example, one of the methods compensates for the LS estimation by computing its asymptotic bias to obtain a consistent identification procedure [23]. If the AR model takes the errors of the previous data into consideration, it can be represented as the errors-in-variance (EIV) model [24]. In the field of geodetic survey, this model has already received extensive attention. The algorithms are used to numerically obtain the total least-square (TLS) solution and the applications of EIV model have been actively investigated [25]. Therefore, the existed studies of the EIV model can be applied in the field of structural damage identification.

This paper is aiming at introducing an AR model which contains additive white noises, and applying a new identification method based on the theory of the partial EIV model to solve the AR model with additive noises. This method allows the consideration of both errors in the current and past observations. The identification method is also applied in a real case. The rest of the paper is structured as follows. In Section 2, the AR models and EIV model are briefly introduced, and then an AR model with additive noises is developed. In Section 3, the theories of LS and TLS are introduced; a TLS solution proposed by Yun et al. [26] for solving the partial EIV model is firstly used to solve the AR model with additive noises; and a more common solution for the partial EIV model considering the possible correlations between the observed vector and design matrix is also proposed. A damage indicator is developed to represent the severity of the damage through a mathematical simulation. In Section 4, the performance of three methods are studied by identifying the damage of a finite element simulation case. Results show that the extended solution considering the possible correlations between the observed vector and design matrix can not only identify the structural damage and degrees but also perform better than the TLS solution proposed by Yun et al. [26] and the LS solution for classical AR model. In the application of the proposed method in the real case, the experimental data of a shaking table test are used to identify the damage of the model of different seismic levels in Section 5.

## 2. AR Model with Additive Noises

The classical AR model is introduced in this section followed by the introduction of the LS solution for the typical AR model. Due to the disadvantages of the LS solution, the AR model with additive noise is developed. 

A classical AR model of order m is described by

(1) $$x_{t}=\beta_{1}x_{t-1}+\beta_{2}x_{t-2}+\ldots +\beta_{m}x_{t-m}+e_{xt},$$

where $x_{t}$ is the discrete-time signal of acceleration responses in this paper; $e_{xt}$ is random noise of $x_{t}$; m is the unknown order of this model and varies from 0 to (t−1); and $\beta_{i}(i=1,2,\cdots ,m)$ is the unknown AR coefficient to be estimated. In a real case, where a civil structure works under operational conditions, noise is usually assumed as Gaussian white noise with a zero-mean and unknown variance, hence the random noise $e_{xt}$ is assumed as the Gaussian white noise in this paper. 

In fact, the left term $x_{t}$ of the model can be regarded as the sum of two terms in the right. The first term is contributed by $x_{t-1}$ to $x_{t-m}$ with unknown coefficients respectively, while the second term represents the noise affection. Denoting $y={\left[x_{t},x_{t-1},\cdots ,x_{t-m+1}\right]}^{T}$, $\beta =[\beta_{1},\beta_{2},\cdots ,\beta_{m}{]}^{T}$, and $e_{y}={\left[e_{xt},e_{x(t-1)},\cdots ,e_{x(t-m+1)}\right]}^{T}$, the AR model can be represented as:

(2) $$y=A\beta +e_{y},$$

where $e_{y}$ is the error corresponding to y. As mentioned before, all of the x are observed. Therefore, both the y and matrix **A** contain errors in real cases. However, the corresponding errors to **A** are omitted unreasonably in the general AR model. Thus, errors should be added to **A** in Equation (2). Meanwhile, taking current observed values and all the errors of previously observed values in consideration, the AR model can be rewritten as,

(3) $$\left[\begin{array}{l} x_{t}\\ x_{t-1}\\ \vdots \\ x_{t-n+1} \end{array}\right]=\left(\left[\begin{array}{l} x_{t-1},x_{t-2},\cdots ,x_{t-m}\\ x_{t-2},x_{t-3},\cdots ,x_{t-m-1}\\ \vdots \\ x_{t-n},x_{t-n-1},\cdots ,x_{t-n+1-m} \end{array}\right]-\left[\begin{array}{l} e_{t-1},e_{t-2},\cdots ,e_{t-m}\\ e_{t-2},e_{t-3},\cdots ,e_{t-m-1}\\ \vdots \\ e_{t-n},e_{t-n-1},\cdots ,e_{t-n+1-m} \end{array}\right]\right)\left[\begin{array}{l} \beta_{1}\\ \beta_{2}\\ \vdots \\ \beta_{m} \end{array}\right]+\left[\begin{array}{l} e_{yt}\\ e_{y(t-1)}\\ \vdots \\ e_{y(t-n+1)} \end{array}\right].$$

That is,

(4) $$y=(A-E_{A})\beta +e_{y},$$

where $E_{A}$ is also assumed as Gaussian white noise. Equation (4) is subject to

(5) $$E\left(e\right)=0,D\left(e\right)=\sigma_{0}^{2}W^{-1},$$

(6) $$W=\left[\begin{array}{cc} W_{y} & W_{yA}\\ W_{Ay} & \omega \end{array}\right],$$

where $W_{y}$ and ω are diagonal weight matrices of y and a. a=vec(A), which is the vector of putting the elements of A into a vector one column after another. $e={\left[e_{y},e_{A}\right]}^{T}$, $e_{A}=\mathrm{vec}\;(E_{A})$. In a special case where the matrix A contains no errors $\left(E_{A}=0\right)$, the model becomes the classical AR model with only noises $e_{y}$. By solving the parameter β of Equation (4), the AR coefficients can be obtained. 

Actually, Equation (4) is the EIV model. As mentioned in the introduction, finding out the solution of the EIV model is quite popular in the field of geodetic survey and various methods have already been developed. Hence the parameters of this AR model with additive noises can be estimated based on the comprehensive literature studies of the EIV model in the next section. 

## 3. TLS Adjustment for the AR Model with Additive Noises

After introducing the AR model with additive noises in Section 2, this section introduces the solutions to the EIV model. Furthermore, the algorithm to estimate the parameters in the AR model with additive noises is presented. 

### 3.1. LS and TLS Method for EIV Model

The simplest approximate approach to estimate unknown vector β is the LS method. The LS method is processed by Lagrange Extremum Method. The estimation results are shown as:

(7) $$\hat{\beta }={\left(A^{T}WA\right)}^{-1}A^{T}Wy.$$

Estimated residual and scalar are:

(8) $$e_{y}=y-A\hat{\beta },$$

(9) $${\hat{\sigma }}_{0}^{2}=\frac{{e_{y}}^{T}We_{y}}{n-m}.$$

However, as noted previously, this method ignores the errors in the design matrix A as if it is deterministic, which may lead bias to the results. To solve this problem, TLS method is proposed to handle both the random observational vector and the random elements in A. The TLS method takes the $E_{A}$ in Equation (4) into consideration, which is surely more rigorous than the LS method. After the first invention of TLS, it is widely used in various fields, and many algorithms such as ordinary TLS and weighted TLS, are used to estimate parameters in the EIV model. Golub and van [27] solved the EIV model by minimizing the Frobenius norm of the corrections of both A and y in 1980, named SVD method. Then, a lot of researches after the SVD method are proposed [28]. The method is shown as follows. 

Firstly, given the estimation criterion function of TLS:

(10) $$S={\left\|D\left[E_{A},e_{y}\right]T\right\|}_{F}.$$

$D=\mathrm{diag}{\left(d_{1},d_{2},\ldots d_{n}\right)}^{T}$; $T=\mathrm{diag}{\left(t_{1},t_{2},\ldots ,t_{m}\right)}^{T}$; and both $d_{i}$ and $t_{i}$ are positive. ${\left\|B\right\|}_{F}$ donates the Frobenius norm of B. Then the minimization object can be solved by computing the singular value decomposition of C:

(11) C=D[A,y]T=UΣV.

where

(12) $$\underset{n,n}{U}=\left[U_{1},\cdots ,U_{n}\right]\in R^{n\times n},$$

(13) $$\underset{m+1,m+1}{V}=\left[V_{1},\cdots ,V_{m+1}\right]\in R^{\left(m+1\right)\times \left(m+1\right)},$$

(14) $$\sum_{n,m+1}=\mathrm{diag}\left(\sigma_{1},\cdots \sigma_{m+1}\right).$$

∑ is the positive diagonal matrix arranged in decreasing order; and $\sigma_{1}\geq \sigma_{2}\geq \ldots \sigma_{m+1}$. When $V_{m+1,m+1}\neq 0$, there is a unique solution:

(15) $$\beta_{TLS}=V_{m+1}/V_{m+1,m+1}.$$

However, Xu et al. [28] pointed out that Pearson’s solution is still a kind of LS method. Furthermore, the SVD method was just a TLS solution based on numerical approximation and was not a real TLS. Therefore, the application of the SVD method is limited even though it is easy.

### 3.2. TLS Solution for the Partial EIV Model

Xu et al. [28,29] extended the EIV model to a more general one named partial EIV model and proposed an algorithm to solve it. In this model, not all the elements of A are random. The partial EIV model is shown as follows:

(16) $$y=\left(\beta^{T}⊗I_{n}\right)\left(h+B\bar{a}\right)+e_{y},$$

(17) $$a=\bar{a}+e_{a},$$

where $\left(h+B\bar{a}\right)=\mathrm{vec}\left(A-E_{A}\right)$; h is a nm×1 deterministic constant vector whose elements consist of non-random elements of $\mathrm{vec}(A-E_{A})$; ⊗ stands for the Kronecker Product. $\bar{a}$ is a t×1 vector with entries of independent random elements in the design matrix, and it is the true value of a; B is a given nt×m matrix that depends on the number of random elements in A; $B\bar{a}$ is a vector representing the random part. The solution for this partial EIV model has also been proposed. 

Firstly, assume that $E_{A}$ and $e_{y}$ are stochastically independent,

(18) $$\mathrm{cov}(e_{y},E_{A})=0,$$

(19) $$W=\left[\begin{array}{cc} W_{y} & W_{yA}\\ W_{Ay} & \omega \end{array}\right]=\left[\begin{array}{cc} W_{y} & 0\\ 0 & \omega \end{array}\right].$$

Then the TLS solution to the partial EIV model is proposed as [28],

(20) $$\hat{\bar{a}}={\left(\omega +S_{\beta }^{T}WS_{\beta }\right)}^{-1}\left\{\omega a-S_{\beta }^{T}W\left(\sum_{i=1}^{m}h_{i}{\hat{\beta }}_{i}\right)+S_{\beta }^{T}Wy\right\},$$

(21) $$\left(N_{h}+N_{B}+N_{Bh}+N_{hB}\right)\hat{\beta }=\mu_{h}+\mu_{B},$$

where $\mu_{h}=\gamma_{h}Wy$, $\mu_{B}=\gamma_{B}Wy$. And

(22) $$h={\left[h_{1},h_{2},\ldots ,h_{m}\right]}^{T},$$

(23) $$B={\left[B_{1},B_{2},\cdots ,B_{m}\right]}^{T}.$$

(24) $$S_{\beta }=\sum_{i=1}^{m}B_{i}{\hat{\beta }}_{i}=\left(\hat{\bar{\beta }}⊗I_{n}\right)B,$$

(25) $$\gamma_{B}={\left[{\hat{\bar{a}}}^{T}B_{1}^{T},{\hat{\bar{a}}}^{T}B_{2}^{T},\cdots ,{\hat{\bar{a}}}^{T}B_{m}^{T}\right]}^{T},$$

(26) $$\gamma_{h}={\left[h_{1}^{T},h_{2}^{T},\cdots h_{m}^{T}\right]}^{T}.$$

$N_{h}$, $N_{B}$, $N_{Bh}$, $N_{hB}$ are m×m matrices, for i,j=1,2,⋯,m, and they are be respectively given by

(27) $$N_{h}(i,j)=h^{T}Wh_{j},$$

(28) $$N_{B}(i,j)={\hat{\bar{a}}}^{T}B_{i}^{T}WB_{j}\hat{\bar{a}},$$

(29) $$N_{Bh}(i,j)={\hat{\bar{a}}}^{T}B_{i}^{T}Wh_{j},$$

(30) $$N_{hB}(i,j)={h_{j}}^{T}WB_{j}\hat{\bar{a}}.$$

Furthermore, Yun et al. [26] proposed an alternative solution to the partial EIV model. The solution is shown as follows:

(31) $$\hat{\beta }={\left({\hat{\bar{A}}}^{T}W\hat{\bar{A}}\right)}^{-1}{\hat{\bar{A}}}^{T}Wy.$$

(32) $$\hat{\bar{a}}=a+\omega^{-1}S_{\beta }^{T}E^{-1}(y-A\hat{\beta }).$$

where $E=W^{-1}+S_{\beta }\omega^{-1}S_{\beta }^{T}$. The final solution can be obtained by iteration. Yun et al. [26] illustrated that the new one could be more compact and direct than the formula proposed by Xu [28], which is easier and can be processed much more quickly if the number of independent random elements of the design matrix A is significantly larger than that of the measurements. It is clear that the EIV model in Equations (16) and (17) and the solutions in Equations (31) and (32) are more general. 

However, in the AR model with additive noises, there should be the same elements in y and A, leading to the same elements existed in $E_{A}$ and $e_{y}$. Therefore, $E_{A}$ and $e_{y}$ may be not stochastically independent. It cannot be simply assumed that $\mathrm{cov}(e_{y},e_{a})=0$, which means the TLS above cannot be used. Here the errors of $x_{i}$ in the $e_{y}$ are not regarded as the same with the errors of $x_{i}$ in the $E_{A}$, i=(t−n+1),⋯t. That is, $e_{yi}$ is not always the same with $e_{i}$, then the method proposed by Yun et al. [26] can be applied to solve the AR model with noises. Even though $e_{yi}$ should be equal to $e_{i}$ in real cases, the real values of the errors in $x_{i}$ are always unknown. Therefore, both $e_{yi}$ and $e_{i}$ have chances to be closer to real errors. Based on this uncertainty, it is more compatible to suppose that $e_{yi}$ and $e_{i}$ are not the same and estimate them independently, than ignoring the errors in matrix A immediately.

As mentioned before, Equation (4) is a special case of the partial EIV model. When t=nm, h=0, Equation (16) turns to Equation (4), which is,

(33) $$y=\left(\beta^{T}⊗I_{n}\right)\cdot Ba+e_{y}=(A-E_{A})\beta +e_{y}.$$

Considering that all the observations in AR model are obtained in the same condition and by the same measuring instrument, the weight of each obtained output time-series signal can be assumed to be the same. Therefore, the diagonal weight matrix W can be simplified as a unit matrix in the AR model in this paper. The TLS parameter estimation steps of the AR model with additive noises are shown as follows [26,28]:

1. Given A and y, $W=I_{(n+1)m}$, h=0;
2. Initialize $\hat{\bar{a}}=a$
3. Compute $\hat{\beta }$ by Equation (31);
4. Compute $\hat{\bar{a}}$ by Equation (32) based on the obtained $\hat{\beta }$ in step 3.
5. Give a predetermined tolerable errors value. Terminate the process if errors between $\hat{\bar{a}}$ and $\hat{\beta }$ are within the given value. Otherwise, go to Step 3.

### 3.3. TLS Solution for the AR Model with Additive Noises

As mentioned above, $\mathrm{cov}(e_{y},e_{a})$ may be not equal to zero, thus the TLS solution for the partial EIV model can be extended to a more general one which does not care about the correlations between $e_{y}$ and $e_{a}$. Firstly, rewrite the EIV model as,

(34) $$\left[\begin{array}{c} y\\ a \end{array}\right]=\left[\begin{array}{c} \left(\beta^{T}⊗I_{n}\right)\left(h+B\bar{a}\right)\\ \bar{a} \end{array}\right]-C_{1}\cdot \bar{e}$$

where $C_{1}\cdot \bar{e}={\left[-e_{y},-e_{a}\right]}^{T}$, and $\bar{e}$ is a s×1 vector which consists of all the real random elements in the vector y and design matrix **A**. $I_{n}$ is an unit matrix with size of n×n. The objective function is then modeled as,

(35) $$\Phi ={\bar{e}}^{T}\bar{W}\bar{e}+2\lambda^{T}(y-\left(\beta^{T}⊗I_{n}\right)\left(h+B\bar{a}\right)+C_{2}\bar{e})$$

where $C_{2}=\left[I_{n}-\left(\beta^{T}⊗I_{n}\right)B\right]\cdot C_{1}$, and $\bar{W}$ is the weight matrix of $\bar{e}$. By differentiating Φ in Equation (26) with respect to $\bar{e},\;\beta$, and λ, and setting all these partial derivatives to zero, we can obtain the following equations:

(36) $$\frac{1}{2}\frac{\partial \Phi }{\partial \bar{e}}=\bar{W}\bar{e}+C_{2}\lambda =0$$

(37) $$\frac{1}{2}\frac{\partial \Phi }{\partial \beta }=-\hat{\bar{A}}\lambda =0$$

(38) $$\frac{1}{2}\frac{\partial \Phi }{\partial \lambda }=y-\left(\beta^{T}⊗I_{n}\right)\left(h+B\bar{a}\right)+C_{2}\bar{e}=0$$

Finally, the general solution can be obtained as,

(39) $$\bar{e}=-{\left(\bar{W}\right)}^{-1}C_{2}^{T}\lambda$$

(40) $$\lambda ={\left(C_{2}{\left(\bar{W}\right)}^{-1}C_{2}^{T}\right)}^{-1}\left(y-\left(\beta^{T}⊗I_{n}\right)\left(h+B\bar{a}\right)\right)$$

(41) $$\hat{\beta }={\left[\hat{\bar{A}}{\left(C_{2}{\left(\bar{W}\right)}^{-1}C_{2}^{T}\right)}^{-1}A\right]}^{-1}{\hat{\bar{A}}}^{T}{\left(C_{2}{\left(\bar{W}\right)}^{-1}C_{2}^{T}\right)}^{-1}y$$

The steps of this TLS algorithm for the partial EIV model are shown as follows:

1. Given **A** and y, denote $W=I_{(n+1)m}$ and h=0
2. Calculate $C_{1}$
3. Compute the LS solution ${\hat{\beta }}_{0}={\left(A^{T}W_{y}A\right)}^{-1}A^{T}W_{y}y$
4. Initialize $C_{2}=\left[I_{n}-\left({{\hat{\beta }}_{0}}^{T}⊗I_{n}\right)B\right]\cdot C_{1}$
5. Obtain ${\hat{\beta }}_{i}$ using Equations (30)–(32) and renew $C_{2}$
6. Provide a predetermined tolerable error value. Terminate the process, if the errors between ${\hat{\beta }}_{i}$ and ${\hat{\beta }}_{i-1}$ are within the given value. Otherwise, go to Step 3 and repeat.

The AIC criterion for the AR model is used in this paper to find out the optimal order m of the AR model, formulated as [30]:

(42) $$\mathrm{AIC}(n)=\ln{\hat{\sigma }}_{a}^{2}(n)+2n/N,$$

where ${\hat{\sigma }}_{a}^{2}$ is the estimated variance of residual errors of order *n*.

The following step concentrates on proving the effectiveness of the TLS method proposed by Yun et al. [26] in the parameter estimation of AR model with additive noises.

### 3.4. Performance Analysis

In this section, a numerical simulation is conducted to study the performance of the two estimating method proposed by Yun et al. [26] in Section 3.2 and extended method in Section 3.3. The estimation results of these two methods under different noises conditions are analyzed carefully, and the comparisons between classical AR model and these two solutions are demonstrated.

Consider the following 4th-order AR model [21],

(43) $$x_{t}=2.4x_{t-1}-3.03x_{t-2}+1.986x_{t-3}-0.6586x_{t-4}+e_{t}.$$

where $e_{t}$ is the white noise with the unknown variance, and $E\left[e^{2}(t)\right]=1,\;e(t)={\left[e_{t},e_{t-1},\cdots \right]}^{T}$. The number of samples is limited to 300, and the signal-to-noise ratio (SNR) is different when applying the noises to the AR model [20].

For each run, the Gaussian white noise is added by the function *awgn* in the MATLAB to all of the output data x. Six conditions of the SNR, 60 dB, 50 dB, 40 dB, 30 dB, 20 dB, and 10 dB, are respectively added to the true value, and the noise series are the same in the same noise condition. The TLS_p_ represents the solution in Section 3.2, and the TLS_E_ represents the solution in Section 3.3.

When n=4, the AIC meets its minimum value. When the AR model is noise-free, all the identification results are equal to the real values $\beta^{T}=[2.4\;-3.03\;1.986\;-0.6586]$. Other results are summarized in Table 1. It can be seen that when the SNR = 60 dB, the solutions of the LS, the TLS_p_ and the TLS_E_ estimation are nearly the same and quite close to the real value, indicating all the methods perform well in this condition. However, the SNR rises as the differences between these two methods increasing. When the SNR is 30 dB, the results of the TLS_E_ method approximate the true value, while both the LS and the TLS_p_ method differ much. When the SNR is 10 dB, the same performances can be found. It is argued that the TLS_E_ solution for the AR model with additive noises can be more accurate. Hence, it may be concluded that even though all the three methods can be used for parameter estimation of the AR model with additive noises, the TLS_E_ estimation method can perform better than the other two. This advantage can be obtained especially in the presence of strong noise in the AR model.

### 3.5. Damage Indicator

After the unknown parameter β of the AR model is obtained, a damage indicator needs to be defined to assess the damage level of the structure. The difference between β in the healthy conditions and that in damage conditions cannot be measured just by visual inspection simply, especially when there is a lot of elements in β. In this paper, the ratio between Euclidean distance of the undamaged β and the damaged β is used to indicate the structural damage. Steps are clarified as follows.

1. Divide the obtained response acceleration data before damage into two parts, i.e., part $A_{0}$ and part $B_{0}$, where $A_{0}$ serves as the baseline data while $B_{0}$ serves as the unknown inspection data to be estimated in the healthy state of the structure.
2. Estimate $\beta_{A0}=[\beta_{A0,1},\beta_{A0,2},\cdots ,\beta_{A0,m}{]}^{T}$ and $\beta_{B0}=[\beta_{B0,1},\beta_{B0,2},\cdots ,\beta_{B0,m}{]}^{T}$. The square of Euclidean distance between $\beta_{A0}$ and $\beta_{B0}$ is determined as,
  (44) $$D_{0}=\sum_{j=1}^{m}\left[(\alpha_{B0,j}-\alpha_{A0,j}\right){]}^{2}.$$
3. Estimate the $\beta_{i}=[\alpha_{i1},\alpha_{i2},\cdots ,\alpha_{im}{]}^{T}$ of the *i*-th response acceleration data after damage. The Euclidean distance between $\beta_{i}$ and $\beta_{B0}$ is calculated as,
  (45) $$D_{i}=\sum_{j=1}^{m}\left[(\alpha_{i,j}-\alpha_{A0,j}\right){]}^{2}.$$
4. Finally, the damage indicator is calculated as the ratio of $D_{i}$ and $D_{0}$,
  (46) $$IF=\frac{D_{i}}{D_{0}}.$$

It is clear that if the data to be estimated are associated with the undamaged structure, the IF is close to 1. Otherwise, the changes of the AR parameters are increasing while the damage rising. Therefore, the IF values are rising as the damage level of the structure is increasing.

Assuming that each β are obtained due to structural damage instead of errors, each estimation result represents a condition of the system. For example, assuming that when SNR = 20 dB, the TLS_p_ solution $\beta^{T}=[2.3563\;-2.9840\;1.9340\;-0.6301]$ in Table 2 is estimated by the output signals of damaged structures in one of the six different conditions by the TLS_p_ method. Then the damage levels of structures should be increasing with the increase of the SNR. The IFs of the example in Table 1 are shown in Table 2.

It can be seen that the IFs are rising as the SNR is increasing, reflecting both the effectiveness of the damage indicator and all the three damage identification methods. As for the same SNR, the IFs based on the TLS_E_ solution for the AR model with additive noises are always smaller than those on TLS_E_ solution for the AR model with additive noises and the LS solution for the classical AR model. For example, when the SNR = 10 dB, the IFs of the LS solution are about 90 times and 134 times as large as the IFs of the TLS_E_ solution. However, when comparing the TLS_p_ solution with the LS solution, in some cases the TLS_p_ solution may be better while in other cases worse than the LS solution. Therefore, both the LS solution, TLS_p_ solution and the TLS_E_ solution for the AR model with additive noises can be effective in this mathematical simulation. However, the former one is not stable enough, and the TLS_E_ solution not only performs the best but also can always obtain accurate results, even in the presence of high amounts of noise.

## 4. Finite Element Simulation

**Figure 1:**
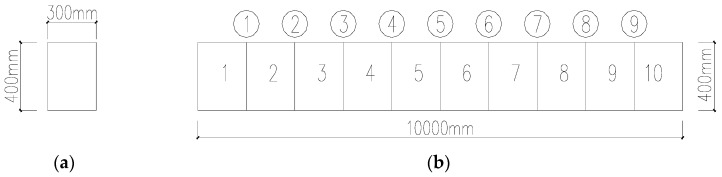
Size of the beam: (**a**) Cross-sectional view of the beam; (**b**) Distribution of the sensors.

The performance of the TLSE solution for the AR model with additive noises is studied by a finite element simulation in this part. The acceleration observations in different damage conditions are used as the time-series signal in the AR model. A simply supported beam with constant cross-section is modeled by the finite element software SAP2000. The size of the model is shown in Figure 1, with a density of 7850 kg/m^3^ and elastic modulus of $2.1\times 10^{11}$ pa. The Gaussian white noise is applied to the beam, and the obtained accelerations of each testing point serve as the response signals in the AR model. Assume that element 4 is damaged into different degrees in four conditions, which are listed in Table 3. The damage of the beam is simulated by reducing the bending stiffness of specific elements in different degrees.

The accelerations of the testing points before damage are used as the undamaged condition. Table 4 lists the first to the fourth structural frequencies under different conditions. It can be seen that when a damage degree of 10% occurs in the element 1, the first order frequency is 9.32 Hz, which is larger than the other conditions, but smaller than the undamaged condition 9.36 Hz. It may be inferred that as for the first-order frequency, the damage of the beam becomes larger as the testing conditions number increasing. 

The first order of the frequency in the undamaged stage is also calculated by frequency calculation formula in Ref. [31]. Firstly, the first order circular frequency can be calculated as,

$$\omega_{1}=\frac{n^{2}\pi^{2}}{l^{2}}\sqrt{\frac{EI}{m}}=\frac{{\left(3.14\right)}^{2}}{{\left(10m\right)}^{2}}\sqrt{\frac{2.1\times 10^{11}N/m^{2}\times 1.6\times 10^{-3}m^{4}}{7850{\mathrm{kg}/m}^{3}\times 0.4m\times 0.3m}}=58.88\;\mathrm{rad}/s,$$

where m is mass; E is the elastic modulus; I is the inertia moment. Hence, the engineering frequency is,

$$f=\omega_{1}/2\pi =9.37\;\mathrm{Hz}$$

**Figure 2:**
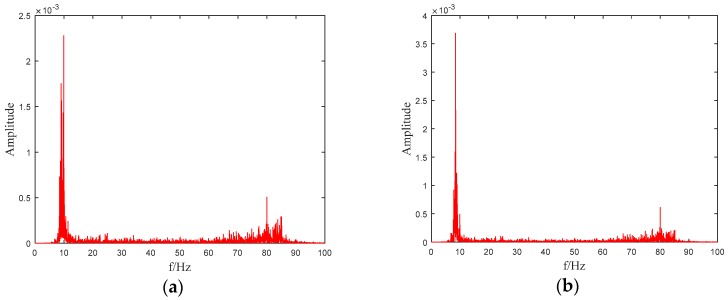
Acceleration power spectral density (PSD) of testing point 5: (**a**) Before damage; (**b**) 50% damage.

The f and $\omega_{1}$ are nearly the same with the simulation results. Since 9.36 Hz is quite close to 9.37 Hz, so the FEM outputs can be assumed to be reasonable and can be used for further study. According to the sampling theorem, sampling frequency is set as 500 Hz, and the total testing time 25 s, respectively. Figure 2 shows the power spectral density (PSD) of the output accelerations of testing point 5. It can be seen that the first order frequency falls into 9–10 Hz in Figure 2a while in Figure 2b the peak is located between 8–9 Hz. The third order frequency of the model beam is about 80 Hz, which can also be inferred in Figure 2a,b. Since the testing point 5 is located in the middle of the beam, there should be no bulges appeared near the second order frequency, which can also be found in the figures. 

After the accelerations of measuring points under different conditions are obtained, the damage indicator presented in Section 3 will be calculated through MATLAB. 

### 4.1. Identification Results in the Condition of No Noises

**Figure 3:**
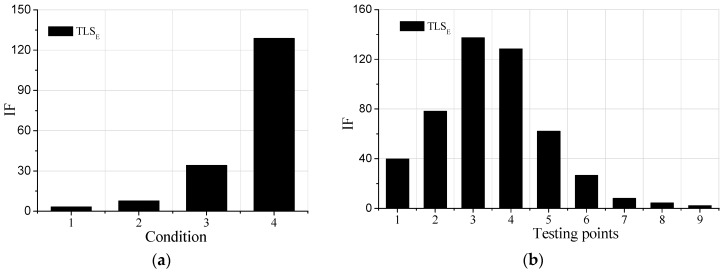
Damage identification results: (**a**) Point 4; (**b**) Condition 4.

Parts of the damage identification results are shown in Figure 3. It can be concluded in Figure 3a that as the damage of element 4 is increasing, the IFs are rising, indicating that the proposed damage detection method can identify the damage degrees in the beam. It can be concluded in Figure 3b that the IFs of point 3 and point 4 are larger than other parts, which means that there is damage in element4. Therefore, the proposed method can also be used for detecting damage locations in structures. However, as for testing points near the undamaged elements, such as point 2, point 3, the IFs are not equal to one. Due to the limited mesh in SAP2000, the damage of element 4 influences its adjacent elements, causing the IFs dropping from testing point 4 to 9. Furthermore, it is clear that the IFs of point 3 and 4, 2and 5, 1 and 7 are not the same, and the points on the left part of element 4 are always larger than the points on the right in the beam, reflecting that the influences of the damage of element 4 may be larger on the left. 

In conclusion, the damage identification method based on the TLSE solution and AR model with additive noises can not only detect damage degrees but also damage locations.

### 4.2. Identification Results in the Condition of Noises

**Figure 4:**
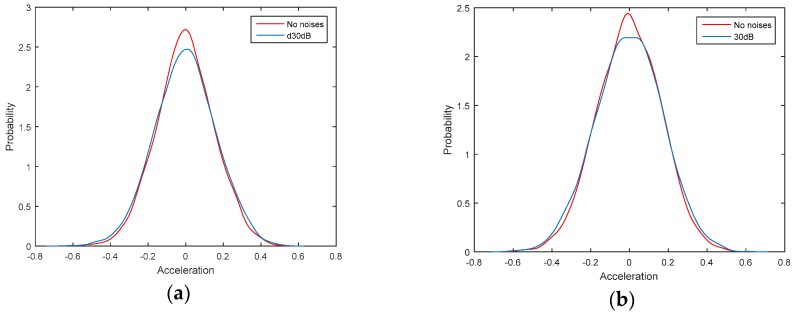
Observed signal with 30 dB Noises: (**a**) Before damaged; (**b**) Condition 3.

In order to study the anti-noises ability of the proposed damage identification method, noises are added to the output signal to simulate the observation errors. Firstly, the accelerations with no noises are identified by the classical AR model and LS solution, which are regarded as the relative standard solution. Then, the accelerations with 30dB noises are identified by the TLS_E_ solution and AR model with additive noises. At last, the accelerations with 30dB noises are also identified by the classical AR model and LS solution for comparing the performances of these two methods. The property density function (PDF) diagrams for the output signal with noises of point 3 is shown in Figure 4. 

**Figure 5:**
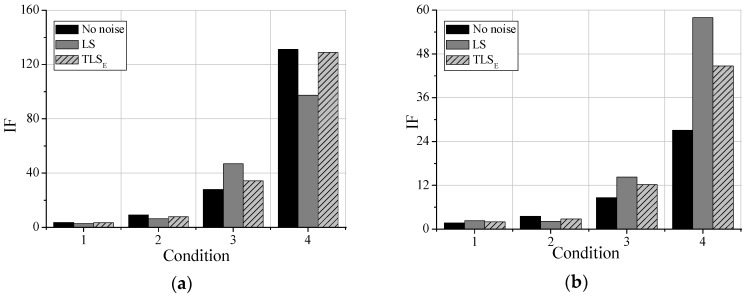
Damage identification results (**a**) Point 4; (**b**) Point 6.

**Figure 6:**
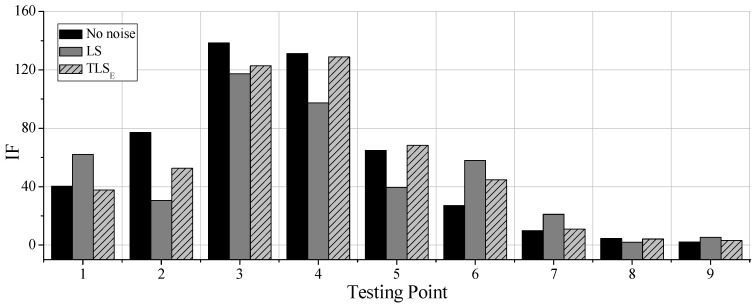
Identification results along the beam in condition 4.

After analyzing all the identification results, it can be concluded that the IF values obtained by the TLS_E_ solution are increasing as the damage of element 4 increasing, reflecting that the damage identification method based on TLS_E_ solution can detect damage degrees. What’s more, IFs obtained by TLS_E_ solution are always closer to the LS solutions in the no noises condition, reflecting that the TLS_E_ solution can identify the damage of structures more accurate when the noises level is 30dB. Parts of results of the testing points are shown in Figure 5. The damage identification results along the beam in condition 4 are shown in Figure 6. IFs of point 3 and 4 obtained by TLS_E_ solution are much bigger than others, reflecting its effectiveness in detecting damage locations even when the observations have noises. However, results obtained by LS are quite different from that of no noises. And the damage location is not quite remarkable, for instance, IFs of point 3 and 4 are not much bigger than the IFs of others, and IF of point 6 is even larger than the IF of point 5. It can be concluded that the LS solution contains bias while the observations have unneglectable noises. 

In conclusion, this finite element simulation can prove that: (1) The IF values obtained by the proposed method are always rising while the damage degrees of the simulated elements are rising. (2) The IFs near the damaged elements are much larger than other IFs. (3) Compared with classical AR model and its LS solution, the IFs obtained by the proposed method in the noisy conditions are always closer to the IFs obtained in the no-noises condition. Therefore, damage identification method based on TLS_E_ solution and AR model with additive noises can detect both structural damage levels and the structural damage distribution, and this proposed method can behave better than the method based on the classical AR model and LS solution.

## 5. Experimental Investigation

In this section, damage levels of a high-rise residential building model due to seismic excitation with increasing intensities are identified using the proposed method qualitatively. Pertinent identification factors (IF) calculated by the proposed and the classical LS method are compared [32].

### 5.1. Experimental Techniques and Results

**Figure 7:**
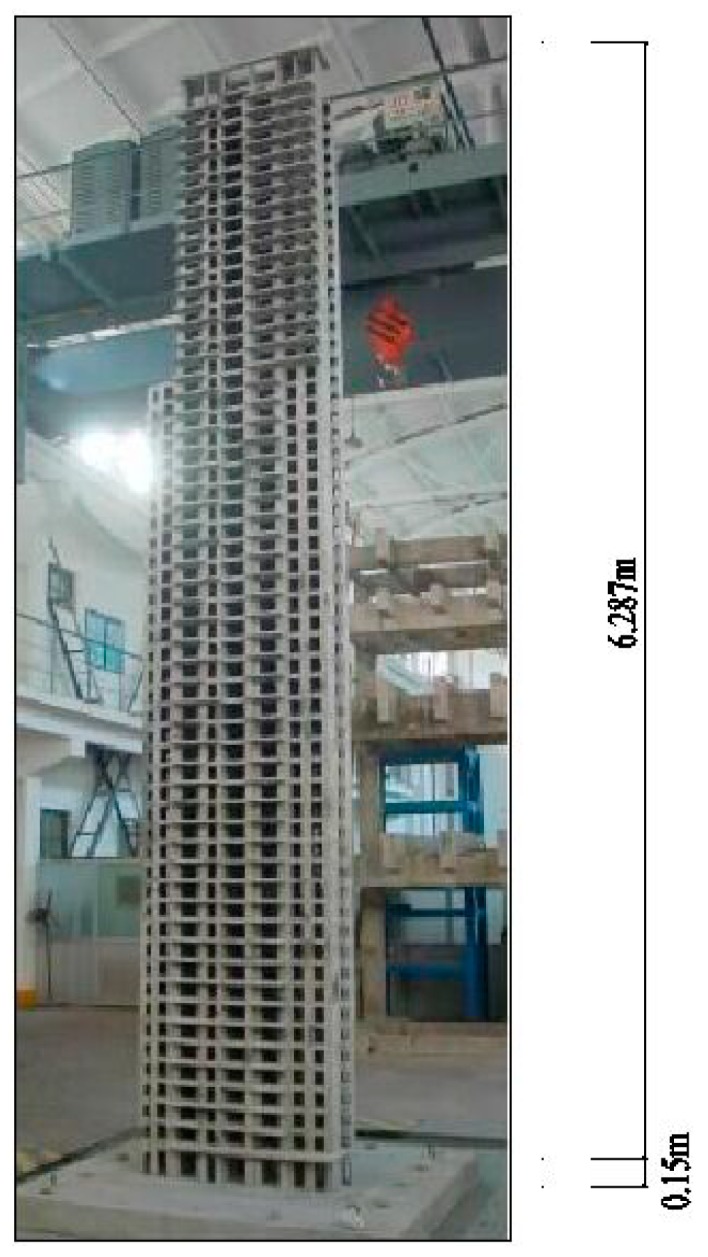
Picture of the model.

Shaking table test is one of the most widely used techniques to assess the seismic performance of structures, including the elastic/inelastic dynamic response of structures [33]. A shaking table test with a 1/30th scale model of a 56-story high-rise building is conducted to investigate the seismic performance. The building is a reinforced concrete structure [34] with a non-regular T-type plane and a height of 179.6 m, which is out of the restrictions specified by the China Technical Specification for Concrete Structures of Tall Building (JGJ3-2010) [35]. Therefore, shaking table is required to assess its seismic performance. The photo of the completed model is shown in Figure 7. 

In this experiment, accelerations and displacements are measured by the dynamic signal acquisition and analysis system DASP2003, developed by Orient Institute of Noise and Vibration. The dynamic strain is obtained by the dynamic and static testing instrument DH3817. 

Seismic excitations, including two natural earthquake records and one artificial record, with increasing intensities adopted in this shaking table test, are shown in Table 5. White noise swiping technique is utilized after each group of seismic excitations to capture the frequency shift due to damage. According to the Chinese Seismic Design Code (2010) [36], “Frequency 6” means ground motion with peak ground acceleration (PGA) 0.018g, “Moderate 6” means ground motion with the PGA 0.05g, whereas “Rare 6” means ground motion with PGA 0.1g. To investigate the hysteresis behavior of the model building, “Rare 7” ground motion with PGA 0.22g is applied. Some dynamic characteristics of the model before and after the earthquake excitation are shown in Table 6. It can be seen that the natural frequency of the model after Frequent 6 and Moderate 6 almost stayed the same, indicating that the damage of the model may be small. While after Rare 6 excitations, the natural frequency reduced 3.9%, reflecting that damage may be increased. After the Rare 7, the nature frequency decreased apparently, possibly due to the significant damage of the model structure. 

**Figure 8:**
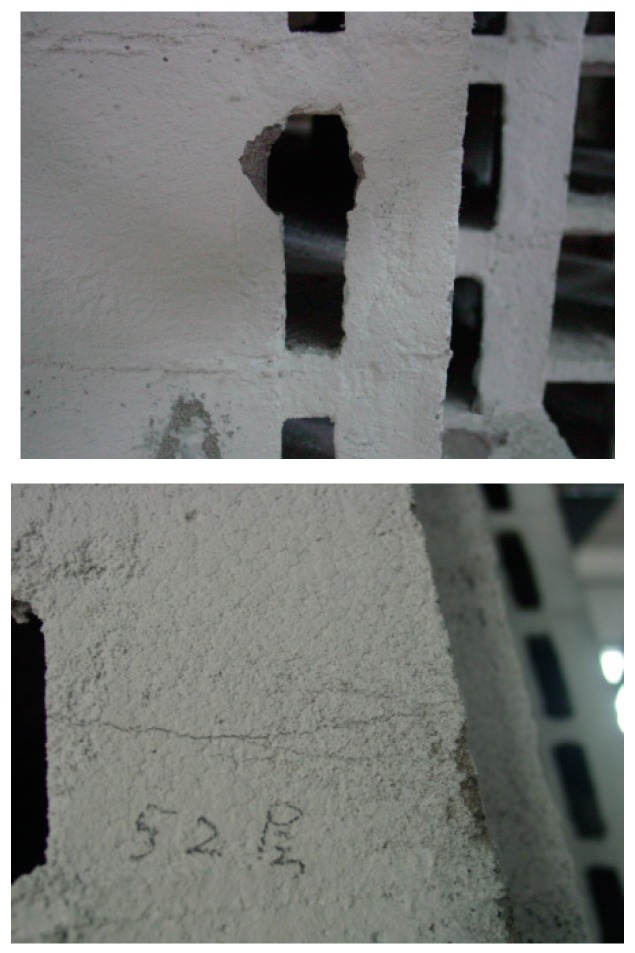
Damage after the test (52nd floor).

It is seen from pertinent results in Ref. [32] that damage level of the building increases significantly with earthquake intensities. Specifically, first, almost no damage occurred after applying the Frequent 6 excitations, indicating the structure remained in the elastic stage. Furthermore, limited damage was captured after the Moderate 6 excitations, showing the structure was repairable. Next, some obvious damage could be observed after applying the Rare 6 excitations. Finally, the model was significantly damaged after severe earthquakes with intensity 7. The behavior of the model building under the considered intensities indicates the structural design satisfies the different levels of seismic performances specified in the codes. However, extensive concrete crakes as well as spalling were observed, especially in the stories higher than the 50th floor, reflecting the whiplash effect is strong. Some remarkable photos of the damaged structure after the test of 52nd floor are shown in Figure 8. It can be seen that there are penetrating cracks and spalling, indicating that the top part of the structure may be damaged significantly after Rare earthquakes of intensity 7. Finite element (FE) simulation [34] of the prototype building is also conducted to validate quantitatively the data obtained by the sensors. 

For simplicity, the experimental results are not introduced here; see Ref. [32] for more details. The acceleration responses of the white noise excitations are used for damage identification in the next section. 

### 5.2. Damage Identification

**Figure 9:**
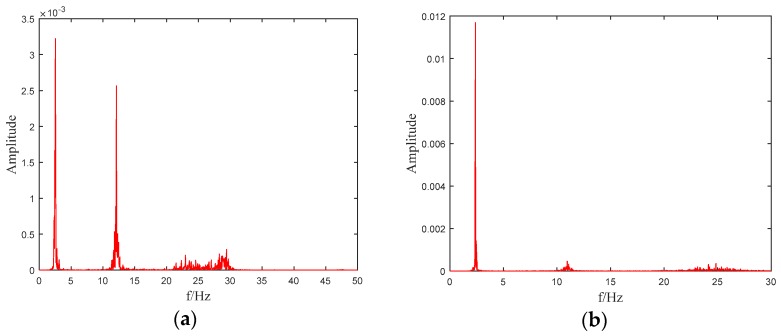
PSD figures for acceleration outputs of the top floor: (**a**) Before the earthquake excitations; (**b**) After the earthquake excitations.

The acceleration sampling frequency of the test is 500 Hz, and 2048 time-series values for each testing point are used in this study. Two of the PSD figures for acceleration outputs of the top floor before and after the earthquake excitations are shown in Figure 9. It can be seen that the PSD of the top floor changes significantly before and after seismic excitations. In Figure 9a there are three peaks, and the first order frequency falls into 2–3 Hz, which can also be seen in Table 6. In Figure 6b there are also three peaks and all the frequencies corresponding to the peak values are relatively smaller than those of Figure 6a, which may be due to structural damages after seismic intensity of Rare 7.

**Figure 10:**
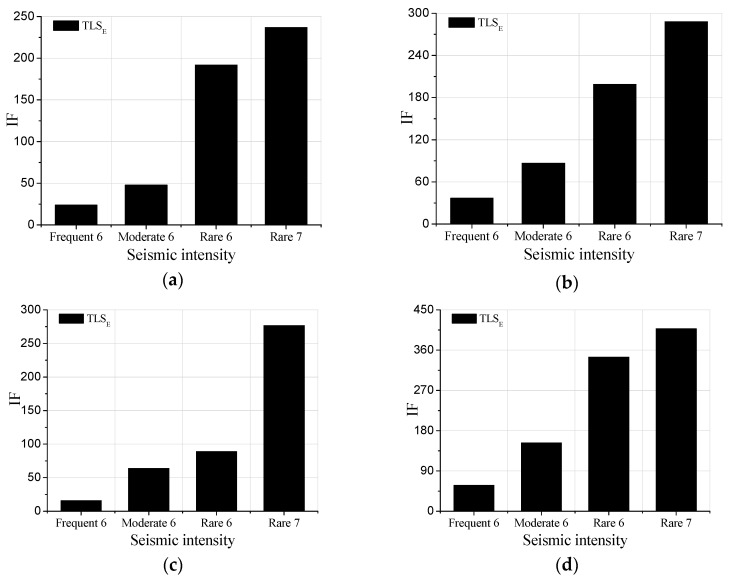
IFs of some floors after different earthquake intensities: (**a**) 8th floor; (**b**) 14th floor; (**c**) 41st floor; (**d**)Top floor.

Figure 10 lists the IF*s* of representative floors calculated by the proposed method in the case of white noise excitations. It can be concluded from Figure 10a–d that for all the stories, the IF*s* become larger as the intensity of earthquake increasing, meaning the damage of the test model increases with intensity. Furthermore, the comparison between Figure 10a–d shows that the IFs of the top story is larger than that of other stories because of the whiplash effect.

**Figure 11:**
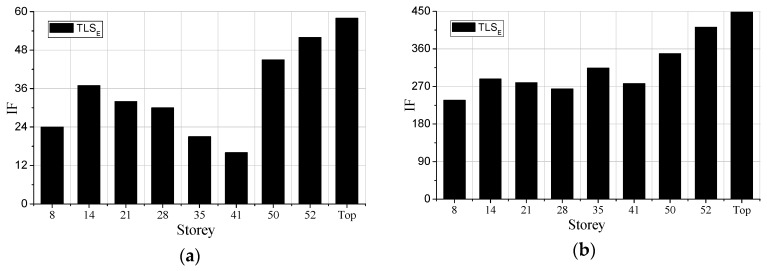
Identification factors (Ifs) along with stories: (**a**) Frequent 6; (**b**) Rare 7.

To further illustrate the distribution of damage levels along with stories, IFs of different floors obtained by the proposed method based on the response acceleration records are shown in Figure 11a,b.

It can be concluded that after applying the Frequent 6 excitation sequence, all the IFs range from 1 to 60, whereas after applying the Rare 7, the IFs increase dramatically. Since the values of IFs are directly associated with damage degree quantitatively, one may argue that the damage degree of the Rare 7 is much larger than that of the Frequency 6. Besides, the damage degrees of 50th, 52nd, and top floors are more substantial than those of the other floors. The damage of 14th, 28th and 8th story is quite significant as well, while the damage of the first story is the smallest. The IFs of the 41st floor is not so large but the IFs of the stories above this floor increase rapidly, indicating that the 41st floor is not seriously damaged compared to the above floors. The preceding damage distribution along with stories are not only limited to the cases shown in Figure 11a,b, but also the same conclusion can be drawn after analyzing all the white noise response data. 

**Figure 12:**
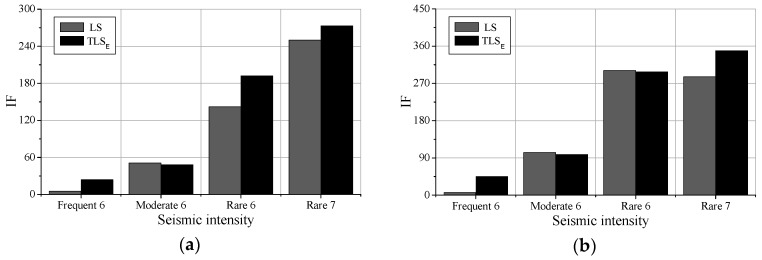
Comparison between least square (LS) solution with total least-squares (TLS_E_) solution: (**a**) 8th story; (**b**) 50th story.

**Figure 13:**
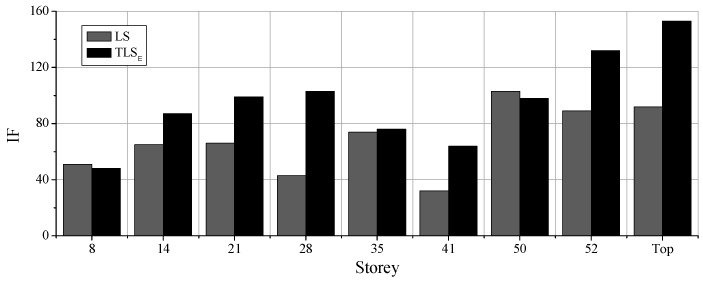
Comparison along with stories after Moderate 6.

The comparisons of the classical AR model solved by LS and the modified AR model solved by the TLS are shown in Figure 12 and Figure 13. Figure 12a,b show the comparisons of the IFs in the 8th floor and the 50th floor after different earthquake intensities. Figure 13 is the comparison of the IFs along with stories after the Moderate 6 excitations. It can be seen from these figures that the results obtained by the two methods do not agree with each other quantitatively. For instance, in Figure 12b, the proposed method gives a higher IF than the LS solution in the case Rare 7, whereas provides a lower IF in the case of Moderate 6. Further, the IFs in Figure 13 of the proposed method demonstrate more visible whiplash effect while the IFs of the traditional AR model are milder. It may argue that the differences between the proposed method and the LS solution shown in Figure 12 and Figure 13 contribute to the later method ignoring errors in the previous time step, which is, illustrated in the preceding sections. 

To summarize, the identification results indicate the usefulness of the proposed method for structural damage detection in real cases. Comparison with the results obtained by the LS solution shows the proposed method is reasonable, at least from a qualitative sense. Besides, the damage degrees and their distribution obtained by the proposed method match well with the corresponding results in Ref. [32], based on analysis of frequency shift and peak displacement/acceleration peak. 

## 6. Conclusions

An Auto-Regressive (AR) model with consideration of additive noises as well as its total least square solution has been presented for structural damage identification in this paper. The total least square method is based on the partial errors-in-variance (EIV) model. The AR model with additive noises takes the errors of all the observed data into consideration. After comparing the proposed method with the classical AR model and least square (LS) solution in a mathematical simulation and a finite element simulation, the advantages of the new identification method have been demonstrated. A damage identification indicator has also been presented to measure the AR parameter differences between healthy stage and the damaged stage, reflecting the damage degrees. Finally, the proposed method has been applied to the acceleration responses of a shaking table test. The proposed method can estimate both the damage level and damage distribution of structures. Hence, the damage estimation method exhibits its usefulness in engineering applications.

This study makes contributes significantly to the literature because the proposed method can reduce the identification errors, and behave well even in high amount of noises, which is more practical than classical identification methods based on the AR models. Furthermore, the achievements in the field of the geodetic surveys are first systematically introduced to the field of vibration-based structural damage identification, which could promote the combination of these two fields to benefit further studies in the field of structural health monitoring.

The simulated beam in Section 4 may be improved to a more real one. Future works may focus on the development of more efficient de-noise methods for structural damage identification. A more general solution of the TLS_p_ for the AR model with additive noises can be studied when considering $\mathrm{cov}(e_{y},e_{a})\neq 0$, especially the weighted TLS solution for putting different kinds of observations together to reduce errors. Other estimation methods and criterion such as BIC, FPF for the EIV model can be introduced into the field of structural health monitoring. 

## Figures and Tables

**Table 1:** Values of parameter estimation.

SNR/dB		NO	60	50	40	30	20	10
LS	$\beta_{1}$	2.4	2.4001	2.3966	2.4067	2.3714	2.3357	2.6494
	$\beta_{2}$	−3.03	−3.0310	−3.0233	−3.0441	−2.9820	−2.9456	−3.5409
	$\beta_{3}$	1.986	1.9862	1.9793	1.9998	1.9452	1.8973	2.4657
	$\beta_{4}$	−0.6586	−0.6587	−0.6557	−0.6624	−0.6486	−0.6162	−0.9030
TLS_p_	$\beta_{1}$	2.4	2.4001	2.3966	2.4070	2.3736	2.3563	2.6931
	$\beta_{2}$	−3.03	−3.0311	−3.0234	−3.0445	−2.9860	−2.9840	−3.6220
	$\beta_{3}$	1.986	1.9861	1.9793	2.0003	1.9491	1.9340	2.5435
	$\beta_{4}$	−0.6586	−0.6588	−0.6557	−0.6626	−0.6500	−0.6301	−1.0326
TLS_E_	$\beta_{1}$	2.4	2.4001	2.3998	2.4010	2.3927	2.3708	2.4715
	$\beta_{2}$	−3.03	−3.0302	−3.0244	−3.0332	−3.0160	−3.0091	−3.1298
	$\beta_{3}$	1.986	1.9861	1.9858	1.9886	1.9742	1.9632	2.1011
	$\beta_{4}$	−0.6586	−0.6587	−0.6566	−0.6600	−0.6521	−0.6433	−0.6757

**Table 2:** IFs of different conditions.

SNR/dB	60	50	40	30	20	10
LS	2.20	2.20 × 10	1.91 × 10^2^	9.80 × 10^2^	4.18 × 10^3^	2.62 × 10^5^
TLS_p_	2.60	2.18 × 10	2.08 × 10^2^	8.20 × 10^2^	1.50 × 10^3^	3.86 × 10^5^
TLS_E_	1.06	7.00	1.99 × 10^1^	8.27 × 10^1^	3.40 × 10^2^	2.86 × 10^3^

**Table 3:** Test conditions (element 4).

Test Condition	Undamaged	1	2	3	4
Damage degree	N	10%	20%	30%	50%

**Table 4:** Structural frequency under different conditions.

Test Condition	Undamaged	1	2	3	4
1st	9.36	9.32	9.27	9.20	8.88
2nd	37.22	37.09	36.94	36.77	35.92
3rd	82.91	82.86	82.79	81.71	80.30
4th	129.30	128.94	128.52	128.04	125.52

**Table 5:** Sequence of the shaking table test.

Test Condition	Sequence Number	Input Seismic Wave
Frequent 6	1	White noise
	2	El-Centro wave, Taft wave, Artificial seismic wave
Moderate 6	3	White noise
	4	El-Centro wave, Taft wave, Artificial seismic wave
Rare 6	5	White noise
	6	El-Centro wave, Taft wave, Artificial seismic wave
	7	White noise
Rare 7	8	El-Centro wave, Taft wave
		White noise

**Table 6:** Dynamic characteristics of the model before and after the test.

Intensity	Test Items	Y			Torsion		X
		1st	2nd	3rd	1st	2nd	1st
Before	Frequency (Hz)	2.54	12.11	29.41	7.62	21.30	3.71
	Period (s)	0.3937	0.0826	0.0340	0.1312	0.0469	0.2695
	Damping ratio (%)	3.25	2.61	2.12	--	--	2.36
Frequent 6	Frequency (Hz)	2.54	12.11	29.21	7.62	21.10	--
	Period (s)	0.3937	0.0826	0.0342	0.1312	0.0474	--
	Damping ratio (%)	4.41	2.71	2.83	--	--	--
Moderate 6	Frequency (Hz)	2.54	11.92	28.72	7.52	20.91	--
	Period (s)	0.3937	0.0839	0.0348	0.1330	0.0478	--
	Damping ratio (%)	4.20	3.04	3.37	--	--	--
Rare 6	Frequency (Hz)	2.44	11.33	27.75	7.30	19.93	--
	Period (s)	0.4098	0.0883	0.0360	0.1370	0.0502	--
	Damping ratio (%)	4.01	3.11	3.35	--	--	--
Rare 7	Frequency (Hz)	2.34	10.75	--	6.84	18.66	--
	Period (s)	0.4274	0.0930	--	0.1462	0.0536	--
	Damping ratio (%)	3.87	3.80	--	--	--	--
